# The effect of pre-service training on post-graduation skill and knowledge retention among mid-level healthcare providers in Mozambique

**DOI:** 10.1186/s12960-015-0011-9

**Published:** 2015-04-16

**Authors:** Caryl Feldacker, Sergio Chicumbe, Martinho Dgedge, Freide Cesar, Gerito Augusto, Molly Robertson, Francisco Mbofana, Gabrielle O’Malley

**Affiliations:** International Training and Education Center for Health (I-TECH), University of Washington, Seattle, WA USA; Department of Global Health, University of Washington, 901 Boren Avenue, Suite 1100, Seattle, 98104 WA USA; National Institute of Health, Mozambique Ministry of Health, Maputo, Mozambique; Department of Human Resources, Mozambique Ministry of Health, Maputo, Mozambique; International Training and Education Center for Health (I-TECH), Maputo, Mozambique

**Keywords:** Healthcare worker training, Pre-service training evaluation, Curriculum revision, Non-physician clinicians, Mozambique

## Abstract

**Background:**

Mozambique suffers from critical shortages of healthcare workers including non-physician clinicians, *Tecnicos de Medicina Geral* (TMGs), who are often senior clinicians in rural health centres. The Mozambique Ministry of Health and the International Training and Education Center for Health, University of Washington, Seattle, revised the national curriculum to improve TMG clinical knowledge and skills. To evaluate the effort, data was collected at graduation and 10 months later from pre-revision (*initial*) and revised curriculum TMGs to determine the following: (1) Did cohorts trained in the revised curriculum score higher on measurements of clinical knowledge, physical exam procedures, and solving clinical case scenarios than those trained in the initial curriculum; (2) Did TMGs in both curricula retain their knowledge over time (from baseline to follow-up); and (3) Did skills and knowledge retention differ over time by curricula? Post-graduation and over time results are presented.

**Methods:**

*t*-tests examine differences in scores between curriculum groups. Univariate and multivariate linear regression models assess curriculum-related, demographic, and workplace factors associated with scores on each of three evaluation methods at the *p* < 0.05 level. Paired *t*-tests examine within-group changes over time. ANOVA models explore differences between Health Training Institutes (HTIs). Generalized estimating equations determine whether change in scores over time differed by curricula.

**Results:**

Mean scores of initial curriculum TMGs at follow-up were 52.7%, 62.6%, and 40.0% on the clinical cases, knowledge test, and physical exam, respectively. Averages were significantly higher among the revised group for clinical cases (60.2%; *p* < 0.001) and physical exam (47.6%; *p* < 0.001). HTI was influential on clinical case and physical exam scores. Between graduation and follow-up, clinical case and physical exam scores decreased significantly for initial curriculum students; clinical case scores increased significantly among revised curriculum TMGs.

**Conclusions:**

Although curriculum revision had limited effect, marginal improvements in the revised group show promise that these TMGs may have increased ability to synthesize clinical information. Weaknesses in curriculum and practicum implementation likely compromised the effect of curriculum revision. An improvement strategy that includes strengthened TMG training, greater attention to pre-service clinical practice, and post-graduation mentoring may be more advantageous than curriculum revision, alone, to improve care provided by TMGs.

## Background

In Mozambique, doctors and other cadres of skilled healthcare workers are in short supply. In 2012, approximately 971 doctors and 10 081 nurses or midwives [1] served Mozambique’s population of an estimated 25 million [2], a provider to patient ratio far below the target density of 2.28 skilled health workers per 1 000 people considered adequate for basic healthcare coverage [3]. Non-physician clinicians, a group of mid-level healthcare workers referred to as *Tecnicos de Medicina Geral* (TMGs), help assuage the effects of the healthcare worker crisis by providing the majority of rural healthcare in Mozambique. TMGs, who are trained in 30 months versus doctors who require 6 years, serve in health units at all levels in the country but are mostly concentrated in rural health centres. TMGs are often the senior clinician in a health facility. In the context of Mozambique’s overtaxed healthcare system and compounded by a growing HIV/AIDS care burden, TMGs may be poorly prepared for the broad requirements of their challenging jobs as frontline primary care providers [4].

The need to generate more TMGs and to provide them with better professional training remains a priority for the Mozambique Ministry of Health (MoH). From 2009 to 2010, the Training Directorate of the MoH with the assistance of the International Training and Education Center for Health (I-TECH), University of Washington, Seattle, revised the TMG curriculum to strengthen the clinical competencies of these mid-level clinicians and to improve post-graduation knowledge retention. The curriculum development process was detailed previously [5]. In brief, the initial curriculum content was primarily organized around discrete subjects, such as anatomy, physiopathology, parasitology, symptomology, laboratory, and pharmacology, whereas the revised curriculum is organized around body systems and blends practical and didactic learning to encourage improved critical thinking and clinical decision-making. The content of the revised curriculum is based upon updated clinical standards for the TMG cadre and reflects the epidemiological profile of the country including emphasis on malaria, malnutrition, HIV, and TB. The final class entering under the initial curriculum entered in 2010 and graduated in December, 2012. All eight health training institutes (HTIs) that educate TMGs across the country now employ only the revised curriculum.

To determine the effect of the curriculum revision on the skills and capacities of the TMGs, an evaluation was conducted by I-TECH beginning in 2010. We compared clinical decision-making, clinical knowledge, and ability to perform a physical exam among 112 TMGs trained under the pre-revision, or *initial*, curriculum to 188 TMGs trained under the *revised* curriculum at two measurement periods: 1) immediately prior to graduation and 2) 10 months after graduation. The first phase of the evaluation, immediately prior to graduation, concluded in July 2013 and found no statistically significant difference between initial and revised curriculum groups across evaluation types: case studies, knowledge test, or physical examination. However, significant differences were discovered between HTIs [6]. At graduation, on average, TMGs of both groups scored below 50% on the physical exam and scored only marginally higher on the clinical case scenarios with an average score of 57%. Students fared better on the knowledge exam, with an average score of 63%.

In this paper, we explore whether the same 112 TMG students from the initial and 188 TMGs students from the revised curriculum maintained their knowledge and skills 10 months post-graduation. We aim to determine (1) At 10 months after job placement, did cohorts trained in the revised curriculum score higher on measurements of clinical knowledge, physical exam procedures, and solving clinical case scenarios than those trained in the initial curriculum? (2) Did TMGs in both curriculum groups retain their knowledge over time (from baseline to follow-up)? and (3) Did skills and knowledge retention over time differ by curricula? As the revised curriculum employed a more systematic approach to the body, increased hands-on learning, and promoted clinical reasoning, it was hypothesized that TMGs trained in the revised curriculum would be better able to strengthen their skills and knowledge once in the workplace in part due to better encoding of knowledge [7]. Thus, if those in the revised curriculum maintained or improved over time in comparison to their initial curriculum peers, it would suggest that their learning experience better prepared them to synthesize their training, translating to improved practice.

## Methods

### Study design

**Figure 1:**
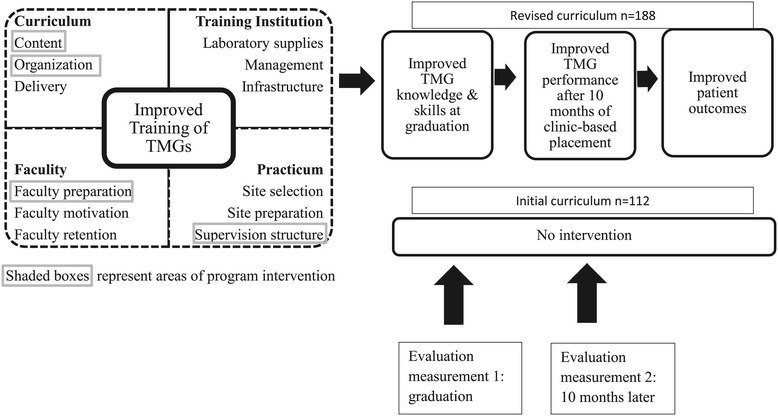
Theoretical model of curriculum revision intervention.

This evaluation used a pre-post, quasi-experimental design to compare clinical knowledge, clinical reasoning, and ability to perform a physical exam by 112 TMGs trained under the initial curriculum with 188 TMGs trained under the revised curriculum at two measurement points: immediately prior to graduation and after 10 months of clinical experience. The study design, outlined in Figure 1, was informed by previous research on evaluation of healthcare worker training [8]. This study reports only the follow-up period.

### Study population

TMG students eligible to graduate from all HTIs between September 2011 and July 2013 were recruited for voluntary enrollment in the study. Measurement at graduation took place in December 2011 in four HTIs that implemented the initial curriculum. Three HTIs graduated students in the revised curriculum in 2012, and an additional four HTIs graduated revised TMGs in July 2013. TMG students only took courses in their specific curriculum; therefore, there were minimal opportunities for contamination between curriculum groups. After graduation, TMGs were assigned to clinical facilities in one of ten provinces or Maputo, the capital city.

### Sample size

The sample size was calculated based on an expected 18% difference in mean scores between the initial and revised curriculum groups, increasing from a hypothesized 75% to 88.5%. Using G*power sample size calculator [9], a total sample size of 280 across 2 groups with 2 measurements for each participant, there is 80% power to detect an effect size of 0.18.

### Assessment methods

The clinical skills and knowledge assessed through these multiple methods was reviewed to reflect the expected TMG performance as established in the MoH-defined competencies for TMGs. All methods were piloted and subsequently revised. Information on the assessment methods was provided in detail previously [6]. In brief, the evaluation of TMGs assessed clinical competency through three methods: 1) case scenarios to assess performance of clinical standards and clinical reasoning, 2) a written exam using both multiple-choice and case-study format to assess clinical knowledge, and 3) a mock physical exam using a standardized checklist and a paid, healthy, physical examination subject. These methods were used at both graduation and follow-up 10 months later.

Case scenarios were created to reflect the scope of practice and expected clinical competencies of TMGs based on MoH guidelines. Cases were developed using clinical standards for five common groups of conditions in Mozambique: suspicion/management of HIV infection, respiratory infections including TB, diarrheal illness, suspicion/treatment of malaria, and emergencies (including obstetric and paediatric emergencies). Twenty-five case studies were developed with the aim of five equivalent scenarios per topic group. Clinical evaluators administered case scenarios orally and recorded answers on a standardized scoring sheet. TMGs were randomized to one clinical scenario within each of the five topical groupings for a total of five case scenarios per data collection round. Case scenarios were implemented for each TMG individually, with the five cases taking approximately 1–2 h. After all five cases were completed, evaluators gave feedback to each TMG, individually, to address TMG weaknesses. To complement the case scenarios, a 100-question, multiple-choice test was developed. The 100-question master test included 20 topics with 5 questions per topic area. Thirty unique knowledge tests were developed from the master by randomly selecting two of the five questions per topic area: each student exam contained 40 questions. The knowledge exam was allotted 60 min. Physical examination skills were tested through a standardized checklist administered during a mock physical exam, excluding genital and rectal exams, with a healthy volunteer who received a nominal fee. Each physical exam was conducted individually in standardized clinic rooms and limited to 15 min. Lastly, at follow-up only, an 11-question, self-administered, workplace survey was implemented that included close-ended questions on clinic type, cadres of coworker, facility equipment, laboratory services, common placement challenges, and ways that they tried to strengthen their skills. No feedback was given after the knowledge test or physical exam.

### Team training and field implementation

I-TECH subcontracted with Mozambique’s National Institute of Health (Instituto Nacional de Saúde, INS), an autonomous technical and scientific institution subordinated to the Ministry of Health. INS responsibilities included collaboration on protocol development, assessment tool drafting and pre-testing, field team recruitment, quantitative and qualitative data collection, field logistics, data entry, data transcription, and data quality assurance in the field. I-TECH responsibilities included data quality assurance, data coding, and data analysis. MoH retains ownership of data.

The INS led a 5-day training prior to each implementation round that included study ethics, study design overview, preparation of field materials, field protocols, and evaluation task training. Two or three medical doctors from provinces where HTIs are located served as clinical evaluators. Practice evaluations and inter-rater exercises were performed during the 5-day training to reach standardization among the evaluators. At graduation, INS teams in each location explained the study, administered informed consent, filled demographic information, and solicited contact information. Participants were assigned the same study number for all evaluation materials at both time periods to reduce potential for bias or data tampering. Names and study numbers were not linked during field implementation or analysis.

Shortly before the 10-month follow-up, participating TMGs in all provinces were contacted, given an evaluation date, and asked to travel to a centrally located evaluation site. Once at the follow-up facility, clinical case scenarios, knowledge tests, and physical exams were administered in the same manner as the initial assessment, using a random selection of cases and test questions for both the clinical cases and knowledge tests. The process of measurement in the provinces took approximately 3 weeks including administrative permissions, study team travel, and participant logistics.

### Data analysis

Quantitative data from the clinical case scenarios, physical exam, and knowledge tests were analyzed using STATA 11.0 [10]. Individual and workplace characteristics were compared between the two curriculum groups using chi-square tests. Scores between curriculum groups 10 months after graduation were compared using *t*-tests. As differences were found between HTIs at baseline, ANOVA models were used to explore inter-HTI differences in mean scores by curriculum group. Univariate linear regression models were used to estimate associations between key factors of interest and assessment scores on each of the three evaluation methods. Independent demographic (gender, age, marital status), curricula (initial or revised), and workplace characteristic (type of clinic, coworkers, clinic characteristics, challenges, and improvement strategies) variables were included in separate models for each exam type. Regression models of case studies and physical exam were adjusted by HTI as scores varied significantly by location. In order to estimate differences in scores adjusted for other factors, variables significant at the *p* < 0.05 level in univariate models were included in multivariate models. Only workplace factors that were significantly associated with at least one exam score at the *p* ≤ 0.05 level are included in the tables to simplify presentation.

To determine whether change in scores over time from graduation to follow-up differed between curricula, generalized estimating equations (GEE) models were utilized using STATA’s xtgee commands to account for correlation in multiple outcomes per person over time, modelling each exam type separately. To adjust for potential confounders, individual characteristics (training group, sex, marital status, and age) were included *a priori* as these factors were found to have a significant association with the outcome in previous analysis. Analysis of case studies and physical exam were adjusted by HTI as scores varied significantly by location. All described models included curriculum group, individual-level factors, time (time0 = graduation; time1 = 10 months post-graduation), and an interaction term between time and curriculum group, to determine whether changes over time differed significantly by curriculum group. For clarity of interpretation, Table 1 describes (a) the effect of time as, “change in score over time” and (b) the effect of the interaction as, “benefit of revised curriculum over time” and includes the calculation of the difference time and interaction (a + b) as, “the overall effect of revised curriculum 10 months post-graduation”.

**Table 1:** **Comparison of demographic characteristics of TMGs in initial versus revised curriculum** ^**a**^

**Characteristics**	**Initial (** ***N*** **= 112)**	**Revised (** ***N*** **= 188)**	***p*** **value**
	**# (%)**	**# (%)**	
Sex			0.29
Male	77 (69.0)	118 (62.8)	
Female	35 (31.0)	70 (37.2)	
Marital status			0.54
Single	86 (77.0)	150 (79.8)	
Married	26 (23.0)	38 (20.2)	
Training institute			-
Chimoio	34 (30.4)	28 (14.9)	
Beira	27 (24.1)	30 (16.0)	
Quelimane	29 (25.9)	27 (14.4)	
Pemba	22 (19.6)	25 (13.3)	
Tete	-	24 (12.8)	
Nampula	-	24 (12.8)	
Chicumbane	-	30 (16.0)	
Age			0.29
Under 25 years	66 (58.9)	99 (52.7)	
25+	46 (41.1)	89 (47.3)	

^a^Results from chi-square tests of comparisons of proportions.

From the previous analysis conducted at baseline, significant variation in case scenario difficulty was found [6]; therefore, analyses of differences in clinical case scores in both cross-sectional and longitudinal models were adjusted by clinical scenario.

### Outcome measures

Outcome variables measured as a percent include 1) continuous variable of average score across the five clinical case scenarios used to assess competency in specific clinical standards; 2) continuous score on the written, multiple choice exam to assess clinical knowledge and reasoning leading to decision-making on patient care for various conditions common to resource-poor countries; and 3) continuous score on the checklist used to assess physical exam skills.

For comparison of means and linear regression models, independent variables of individual characteristics include the following: 1) curriculum type (initial versus revised), 2) HTI location, and 3) demographic variables including age, marital status, and gender. Independent variables of workplace characteristics include the following: clinic type, co-worker mix, and characteristics such as whether the clinic provided in-patient care, had internet access, served as a reference hospital, available diagnostics, laboratory services, workplace challenges, and improvement strategies used to gain skills.

### Ethics statement

The study was approved by both the Centers for Disease Control in Atlanta, Georgia, USA, and Mozambique’s National Committee for Health Bioethics (protocol #5741/002, 27 July 2011). The internal review board of the University of Washington determined that this evaluation was not a human subjects research but a routine programme evaluation, granting a non-research determination. As part of the approved protocol, a comprehensive study consent form was read to all potential TMG participants, and participation in both study rounds was voluntary.

## Results

### Demographics and workplace characteristics of TMGs

Demographics of the 112 and 188 TMGs trained in the initial and revised curriculum are presented in Table 1. One TMG from the baseline was not located for follow-up; all other 112 TMGs from the initial curriculum and 188 from the revised curriculum were included. No TMG refused participation in either round. The number and proportion of TMGs from each HTI are presented: four schools had separate students trained in both curricula (Chimoio, Beira, Quelimane, Pemba) while three additional schools (Tete, Nampula, Chicumbane) had students only from the revised. There are no significant differences in sex, marital status, and age between curriculum groups. The majority of workplace characteristics were similar between groups (Table 2).

**Table 2:** **Comparison of workplace characteristics of TMGs in initial versus revised curriculum** ^**a**^

**Workplace characteristics**	**Initial (** ***N*** **= 112)**	**Revised (** ***N*** **= 188)**	***p*** **value**
	**# (%)**	**# (%)**	
Clinic type			0.69
Health Centre I	24 (21.4)	45 (23.9)	
Health Centre II	63 (56.3)	96 (51.1)	
Hospital (all)	25 (22.3)	47 (25.0)	
Coworkers			
Physician	60 (53.6)	98 (52.1)	0.81
Clinical officer	64 (57.1)	102 (54.3)	0.63
Nurse	110 (98.2)	187 (99.5)	0.29
Pharmacy staff	81 (72.3)	116 (61.7)	0.06
Laboratory staff	61 (54.5)	63 (33.5)	0.00
Clinic characteristics			
In-patient care	57 (50.9)	98 (52.1)	0.84
Internet access	22 (19.6)	28 (14.9)	0.27
Reference hospital	43 (38.4)	93 (49.5)	0.06
X-ray available	30 (26.8)	34 (18.1)	0.08
Has TB smear microscopy	78 (69.6)	122 (64.9)	0.40
Available rapid tests	98 (87.5)	184 (97.9)	0.00
No on-site laboratory	21 (18.8)	57 (30.3)	0.03
Workplace challenges			
Poor supervision	16 (14.3)	23 (12.2)	0.61
Poor pre-service training	6 (5.4)	15 (7.9)	0.39
Differences between classroom and work place	39 (34.8)	57 (30.3)	0.42
Missing clinic equipment	85 (75.9)	149 (79.3)	0.50
Little access to diagnostic equipment	58 (51.8)	122 (64.9)	0.03
Missing guidelines for complex patients	22 (19.6)	51 (27.1)	0.14
Workload	68 (60.7)	127 (67.6)	0.23
Improvement strategies			
Received clinical supervision	92 (82.1)	167 (88.8)	0.10
Consult guidelines or protocols	75 (67.0)	120 (63.8)	0.58
Search the Internet	35 (31.3)	43 (22.9)	0.11
Request supervisor’s help	42 (37.5)	66 (35.1)	0.68
Ask work colleague for help	83 (74.1)	157 (83.5)	0.05
Refer patients to other clinic	82 (73.2)	147 (78.2)	0.33

^a^Numbers and percentages reflect only affirmative answers within each curriculum group. *P* values are from results from chi-square comparison of proportions for each data row.

### Aim 1: comparison of assessment scores between curriculum groups

Mean scores on the exams at follow-up differed significantly between groups on two of the three exam types (Table 3). Of those TMGs trained in the initial curriculum, mean scores 10 months post-graduation were 52.7%, 62.6%, and 40.0% on the clinical cases, knowledge test, and physical exam, respectively. Mean scores for the revised curriculum TMGs at graduation were 60.2%, 62.0%, and 47.6% on the clinical cases, knowledge test, and physical exam, respectively. Revised curriculum scores were significantly higher than the scores of the initial group on the clinical cases (*p* < 0.001) and physical exam (*p* < 0.001), but not on the knowledge test. Additional tests of differences in means between HTIs on each of the exam types found scores differ significantly by training institute (not shown).

**Table 3:** **Comparison of mean assessment scores** ^**a**^ **between curricula 10 months post-graduation**

	**Initial (** ***N*** **= 112)**	**Revised (** ***N*** **= 188)**	***p*** **value**
Clinical cases overall			<0.0001
Mean	52.7	60.2	
IQR	46.9–59.2	49.8–69.9	
SD	10.4	13.8	
Knowledge tests			0.62
Mean	62.6	62.0	
IQR	57.5–71.2	52.5–70	
SD	11.2	11.0	
Physical exam			<0.0001
Mean	40.0	47.6	
IQR	30.2–48.8	37.5–56.7	
SD	12.6	12.9	

IQR: Inter-quartile range; SD: standard deviation.

^a^Results of *t*-tests.

### Factors that influence assessment scores at follow-up

Results from both univariate and multivariate linear regression models of factors that influence continuous scores on all three assessment types 10 months post-graduation are presented in Table 4. Analysis of clinical case scores was adjusted to include the specific clinical cases each TMG received. HTI was included in multivariate models of clinical cases and physical exam to adjust for inter-school differences. In univariate models of clinical case scores, several variables demonstrate significance. First, students in the revised group (7.14; CI: 4.23, 10.04) and younger students (3.09; CI: 0.18, 6.00) scored significantly higher than those in the initial curriculum or older students. Among workplace characteristics, those in Health Centre II (district or rural hospitals) (−4.50; CI: −8.19, −0.80) scored lower than those in Health Centre I (rural health centres) while those students who worked in clinics with a doctor (4.16; CI: 1.24, 7.07), clinical officer (4.40; CI: 1.46, 7.34), or pharmacy workers (3.65; CI: 0.58, 6.73) scored higher than students who did not work with that cadre of coworker. Other workplace characteristics were also influential. Students who worked in clinics that provided in-patient care (3.30; CI: 0.36, 6.23), served at referral hospitals (6.20; CI: 3.34, 9.06), had X-rays (5.07; CI: 1.45, 8.69), and had TB smear microscopy (6.47; CI: 3.39, 9.55) did better than their peers whose placements did not have those attributes. Students in clinics with no on-site laboratory did worse (−3.82; CI: −7.23, 0.41) than those placed in clinics with lab services. In the multivariate model, adjusted for HTI, individual characteristics including revised curriculum (4.58; CI: 1.54, 7.61) and younger age (3.42; CI: 0.85, 5.98) students retained their advantage over initial curriculum and older students. Those students whose workplace had TB smear microscopy available still scored higher than their peers (6.27; CI: 1.35, 11.19) while those placed in Health Centre II (−3.42; CI: −6.85, 0.01) still scored lower than those placed in Health Centre I.

**Table 4:** **Estimated effect of key factors on mean assessment scores at follow-up**

**Characteristics**	**Clinical cases** ^**a,b**^		**Knowledge test**		**Physical exam** ^**b**^	
	**Univariate**	**Multivariate**	**Univariate**	**Multivariate**	**Univariate**	**Multivariate**
Individual characteristics						
Training						
Revised	7.14***	4.58**	−0.66		7.61***	5.60***
	(4.23, 10.04)	(1.54, 7.61)	(−3.26, 1.95)		(4.59, 10.62)	(2.85, 9.14)
Sex						
Male	0.47		4.48***	4.41***	4.66**	5.42***
	(−2.65, 3.58)		(1.90, 7.08)	(1.78, 7.03)	(1.54, 7.80)	(2.41, 8.42)
Marital status						
Single	3.52		3.39*	0.44	4.41*	1.16
	(−0.09, 7.12)		(0.34–6.44)	(−2.74, 3.63)	(0.74–8.07)	(−2.36, 4.68)
Age						
Under 25	3.09*	3.42**	4.98***	4.89***	1.89	
	(0.18, 6.00)	(0.85–5.98)	(2.51, 7.45)	(2.35, 7.43)	(−1.15, 4.93)	
Workplace characteristics						
Clinic type						
Health Centre I	---	---	---		---	---
Health Centre II	−4.50*	−3.42*	0.00		−4.29*	−2.73
	(−8.19, −0.80)	(−6.85, 0.01)	(−3.15, 3.15)		(−8.06, −0.54)	(−6.21, 0.74)
Hospital (all)	0.07	−3.41	−0.65		−2.33	−2.93
	(−4.19, 4.34)	(−8.01, 1.19)	(−4.34, 3.03)		(−6.72, 2.06)	(−7.17, 1.32)
Coworkers						
Has physician	4.16**	−2.55	−0.70		2.73	
	(1.24, 7.07)	(−7.78, 2.69)	(−3.22, 1.81)		(−0.29, 5.76)	
Has clinical officer	4.40**	1.84	−0.41		1.94	
	(1.46, 7.34)	(−2.44, 6.11)	(−2.94, 2.13)		(−1.10, 4.98)	
Has pharmacy worker	3.65*	−1.50	−0.24		−0.73	
	(0.58, 6.73)	(−6.76, 3.75)	(−2.90, 2.42)		(−2.46, 3.92)	
Clinic characteristics						
In-patient care	3.30*	−2.12	2.05		1.70	
	(0.36–6.23)	(−6.15, 1.91)	(−0.45, 4.57)		(−1.32, 4.73)	
Referral hospital	6.20***	3.26	0.85		4.12**	3.81*
	(3.34, 9.06)	(−0.78, 7.31)	(−1.68, 3.38)		(1.12, 7.14)	(0.44, 7.18)
X-ray available	5.07**	3.19	−0.46		1.00	
	(1.45, 8.69)	(−0.99, 7.36)	(−3.54, 2.61)		(−2.70, 4.70)	
Has TB smear microscopy	6.47***	6.27**	0.34		1.33	
	(3.39–9.55)	(1.35, 11.19)	(−2.33, 3.01)		(−1.88, 4.54)	
No on-site laboratory	−3.82*	0.34	1.05		−0.23	
	(−7.23, −0.41)	(−4.05, 4.72)	(−1.82, 3.93)		(−3.69, 3.23)	

Results from linear regression. 95% CI in parenthesis.

*p ≤ .05; **p ≤ 0.01; ***p ≤ .0.001. ^a^Adjusted for specific clinical case received. ^b^Multivariate models for clinical case and physical exam were adjusted by HTI.

For the knowledge test, men (4.48; CI: 1.90, 7.08), singles (3.39; CI: 0.34, 6.44), and younger students (4.98; CI: 2.51, 7.45) scored significantly higher than women, married, or older students, respectively. The positive association between both male and younger age on knowledge score remains similar and significant in multivariate analysis.

On the physical exam, students in the revised curriculum (7.61; CI: 4.59, 10.62), men (4.66; CI: 1.54, 7.80), and singles (4.41; CI: 0.74, 8.07) scored higher than those in the initial curriculum, female, and married student peers. Among workplace factors, those in Health Centre II (−4.29; CI: −8.06, −0.54) scored lower than those placed in type I facilities while those at referral hospitals did better (4.12; CI: 1.12, 7.14) than their peers. In the multivariate model, adjusted for HTI, individual characteristics including revised curriculum group (5.60; CI: 2.85, 9.14) and men (5.42; CI: 2.41, 8.42) outperformed their initial curriculum and female peers. Placement in a referral hospital placement increased scores (3.81; CI: 0.44, 7.18).

### Aim 2: within-curriculum group changes in mean assessment scores over time

Between graduation and 10 months later, mean scores changed significantly for both curriculum groups. Table 5 shows results from within-group comparisons over time. Among the initial curriculum group, clinical case scores decreased significantly from an average of 56.7% to 52.7% (*p* = 0.01) while physical exam scores decreased from 49.1% to 40.0% (*p* < 0.0001) between graduation and 10 months follow-up. There was no significant change in knowledge score between time periods. Among the revised curriculum group, clinical case scores increased significantly from an average of 57.3% to 60.2% (*p* = 0.008). There was no significant change in physical exam or knowledge score over time for those in the revised group.

**Table 5:** **Within-curriculum group comparison of mean assessment scores** ^**a**^ **for TMGs over time**

	**Graduation**	**10 months follow-up**	***p*** **value**
Initial curriculum			
Clinical cases overall			
Mean	56.7	52.7	0.01
95% CI	54.0–59.3	50.7–54.6	
SD	13.9	10.4	
Knowledge tests			
Mean	63.5	62.6	0.40
95% CI	61.5–65.5	60.5–64.7	
SD	10.7	11.2	
Physical exam			
Mean	49.1	40.0	<0.0001
95% CI	46.5–51.9	37.6–42.4	
SD	14.3	12.6	
Revised curriculum			
Clinical cases overall			0.008
Mean	57.3	60.2	
95% CI	55.5–59.1	58.2–62.2	
SD	12.7	13.8	
Knowledge tests			0.42
Mean	62.6	62.0	
95% CI	60.9–64.3	60.4–63.5	
SD	11.8	11.0	
Physical exam			0.07
Mean	49.7	47.6	
95% CI	47.9–51.4	45.7–49.5	
SD	12.3	12.9	

CI: confidence interval. SD: standard deviation.

^a^Results of paired *t*-test of scores.

### Aim 3: factors that influence skills and knowledge retention over time

Lastly, Table 6 illustrates the effect of individual-level factors on the three exam scores over time, adjusted for HTI. For the clinical cases, those under age 25 scored an average of 2.36% higher (CI: 0.30, 4.42) than older students averaged over both time points. Clinical case scores overall decreased an average of 3.85% (CI: −6.51, −1.18) over time. However, the revised group increased an additional 6.71% over time (CI: 3.32, 10.10), meaning that the revised group scored 2.86% higher, on average, at follow-up than their peers. For the knowledge test, men scored on average 4.21% (CI: 2.01, 6.41) higher than their female peers while younger students scored 5.39% (CI: 3.21, 7.58) higher than older students overall. No significant change in time or by curriculum group was found. For the physical exam, men scored an average of 3.47% higher (CI: 1.37, 5.57) than women at both time periods. Those under 25 also outperformed their older peers by 2.44% on average (CI: 0.33, 4.56). Between graduation and follow-up, physical exam scores decreased an average of 9.18% (CI: −12.39, −5.97); however, those in the revised group regained an average of 7.12% over time (CI: 3.21, 11.02), signifying an overall reduction of 2.06%.

**Table 6:** **The estimated effect of key factors on the mean difference of assessment scores over time (** ***N*** **= 600)**

**Individual characteristics**	**Clinical cases** ^**a,b**^	**Knowledge test**	**Physical exam** ^**b**^
Training			
Revised	−0.77	−0.30	0.40
	(−3.77, 2.22)	(−2.79, 2.19)	(−2.63, 3.45)
Sex			
Male	1.35	4.21***	3.47***
	(−0.82, 3.52)	(2.01, 6.41)	(1.37, 5.57)
Marital status			
Single	1.37	1.23	1.46
	(−1.20, 3.96)	(−1.49, 3.97)	(−1.19, 4.11)
Age			
Under 25	2.36*	5.39***	2.44*
	(0.30, 4.42)	(3.21, 7.58)	(0.33, 4.56)
Change in score over time (a)	−3.85**	−0.86	−9.18***
	(−6.51, −1.18)	(−2.84, 1.12)	(−12.39, −5.97)
Benefit of revised curriculum over time (b)	6.71***	0.20	7.12***
	(3.32, 10.10)	(−2.35, 2.75)	(3.21, 11.02)
Overall change in score of TMGs in revised group 10 months post-graduation (a + b)	+2.86	−0.66	−2.06

Results from multivariate generalized estimating equations. 95% CI in parenthesis.

*p ≤ 0.05; **p ≤ 0.01; ***p ≤ 0.001. ^a^Adjusted for specific clinical case received. ^b^Multivariate models for clinical case and physical exam were adjusted by HTI.

## Discussion

Although I-TECH and the MoH attempted to strengthen the TMG pre-service curriculum and improve the overall quality of health services in the country, the effort produced largely disappointing results. Similar to the baseline results reported previously [6] and to similar research on healthcare worker capacity elsewhere [11-13], neither group of TMGs scored as high 10 months post-graduation nor demonstrated acquisition of skills over time as anticipated at study design. However, there are several positive outcomes evidenced by the results. TMGs in the revised curriculum did score an average of 7% higher on both the clinical cases and the physical exam than their peers in the initial curriculum, a small but significant improvement. For the clinical cases, TMGs in the revised group and those with access to diagnostic equipment scored higher than their fellow TMGs. HTI had a significant, and varied, effect on scores as well. For the physical exam, the revised group and TMGs from specific types of health centres outperformed their peers; HTI, again, played an influential role. Between graduation and 10 months later, TMGs trained in the revised curriculum retained their skills and knowledge better than their peers trained in the initial curriculum. Among students trained in the initial curriculum, clinical case and physical exam scores decreased significantly; knowledge scores held constant. For the revised group, clinical case scores actually improved slightly over time; physical exam and knowledge test scores remained largely stable. The apparent benefit of the revised curriculum over the initial curriculum on TMG assessment scores increased over time.

The small, but sustained, improvement of the revised group does show some promise on the positive effects of the revised curriculum on healthcare worker training. Revised group TMGs may have been better prepared for success in the workplace [14] and to retain their skills over time [15] as a result of more practice-intensive, experiential-based learning, enhancing their ability to learn on the job [16]. Additionally, the systems-based organization of the new curriculum likely contributed to a well-organized knowledge base which improves both knowledge application and retention [17]. Moreover, as the positive trend illustrated in these results was evidenced by the first group of TMGs trained by the new curriculum, additional years of implementation experience at the HTIs and their faculty could solidify the effects of the revised curriculum on the TMG performance and lead to continued significant improvement of TMGs over time. If this is true, as our results suggest, future evaluations of training programmes may need to allow for additional time between initial implementation and evaluation to allow time for delayed effect of the intervention.

Even if properly trained, TMGs may encounter challenges putting their knowledge into practice in the workplace, which may negatively impact knowledge retention and strengthening of clinical reasoning [18]. Fewer than 30% of both groups were placed in facilities with an on-site lab or capacity for X-rays, limiting their experience with these diagnostic tools, and over 75% of both groups reported that they were challenged by missing key clinical equipment. Although the vast majority of both groups had access to rapid tests, the lack of other laboratory and diagnostic services may decrease opportunities to correctly diagnose and treat patients based on evidence. To overcome workplace deficits, TMGs from both curricula appear eager to learn from their peers: more than 82% of TMGs reported receiving clinical supervision and almost three quarters seek assistance from a colleague when needing clinical advice.

To systematically address workplace gaps, continuing education and improved supervision may help mitigate the limitations of pre-service training to ensure healthcare workers deliver quality HIV services. Health worker motivation is an important component of health worker performance [19,20]. In previous studies on healthcare worker motivation, continuing education was reported among key factors that aided job retention and job satisfaction [21-23]. Although internet access is low among all TMGs (<20%), creating opportunities for continuing education for TMGs, potentially using mHealth initiatives on mobile phones [24], might prove appropriate to improve the quality of care provided by TMGs in the future. Also, although health worker training, alone, might not be effective, training plus supervision strengthening is likely to improve healthcare worker performance, motivation, and satisfaction [20]. Additional workplace-based observation and assessment combined with supportive feedback provided by well-trained mentors might also help TMGs continue to gain skill and confidence [25,26]. With much responsibility placed on TMGs to provide the majority of primary care in rural areas, increased effort to implement workplace-based strategies that further train, mentor, and motivate TMGs seems timely and necessary.

Several results from this evaluation provide insights for others hoping to strengthen pre-service training. First, implementation fidelity can greatly influence an intervention’s success [27]. Although the revised curriculum was intended to be implemented in its entirety, variations in programme implementation such as faculty preparation or facility differences are common and may negatively influence intervention implementation [28]. Additionally, preparation for the curriculum intervention may have been inadequate. Recent experience from a programme in Tanzania to improve professional healthcare worker training through curriculum revision and improved faculty training required 18 months of intensive, comprehensive intervention [29,30]. A similarly-intensive, 9-year effort in Rwanda also successfully improved medical education, on-the-job performance, and key health indicators with an investment of approximately $15 million annually [31]. In contrast, in Mozambique, few HTI faculty were involved in the 3-year curriculum revision process, and teacher training was largely limited to a 3-day workshop aimed at improving teaching skills in hands-on, practical learning. As compared to other successful efforts, the faculty participation and preparation may have been inadequate to engage or train the teachers effectively for the demands of the revised curriculum.

There are several limitations that affected the evaluation implementation. First, as noted previously, the level of clinical cases varied significantly within topic groups. Although randomization meant that each student had an equal likelihood of getting a hard or easy case, the variation may have put some TMGs at a disadvantage. Also, the materials available to the TMGs to conduct the mock physical exam differed between and within schools, a bias that we cannot quantify. Furthermore, the study was not designed or powered to examine inter-HTI differences quantitatively, and we were not able to include initial curriculum students from all HTIs, limiting our ability to completely attribute differences to the effect of the curriculum as opposed to differences in schools. Lastly, the lack of effect between curriculum groups at follow-up or over time may be explained by Type III error or failure to implement the revised curriculum programme as intended.

Overall, this evaluation has several strengths. Primarily, to the best of our knowledge, this is the first standardized, longitudinal evaluation of this cadre of healthcare worker training in Mozambique. The two time points of data collection also provide insight into knowledge retention while the triangulation of assessment data provides a more robust picture of TMG capacity. Moreover, the process of developing and implementing the evaluation was conducted in partnership with the MoH, which may help translate the findings more rapidly into policy changes at the national or HTI level. Additionally, the implementation of the study helped increase local capacity, improving the skills of the research team to conduct rigorous assessments of healthcare worker competency which may be applied to other cadres in Mozambique. Sharing the limitations of the evaluation design and implementation may also help others further strengthen evaluation of health training programmes in the future.

## Conclusions

Results from the study were shared through an interactive workshop with key stakeholders in Mozambique including representatives from all HTIs, INS staff, MoH officials, local partner organizations, and the study team. Several recommendations were offered to further strengthen TMGs during training and post-graduation. First, interventions are needed to improve faculty quality including specific efforts to hire full-time, highly qualified faculty members dedicated to teaching TMGs and support additional training and mentoring to increase the effectiveness of existing faculty. Second, the internship components of the TMG course must be improved. Noted interventions included ensuring that TMG faculty from the HTIs accompanied TMGs in the placement sites to provide additional quality control and supervision, reduce the tutor to student ratio to enable more students to practice skills rather than observe, and changing the format of the internship to allow for more intensive, clinical immersion in each topic area. Lastly, although HTIs have varying resources, all HTIs would benefit from additional financial investment to increase the capacity of the practice labs, provide sufficient quantities of training materials, and hire sufficient HTI faculty and internship tutors to strengthen implementation of the curriculum as designed.

Overall, it is obvious that the need for TMGs is great while the preparation of, and support for, TMGs may still be inadequate. We believe that sharing the results of this study helps demonstrate the importance of comprehensive efforts to strengthen health worker training, recognizing that improvements in the curriculum, alone, are unlikely to produce the desired changes. An approach to strengthening human resources for health should aim for an equal mix of increasing the workforce numbers, increasing the overall competence of those trained, and improving worker motivation to improve performance of the healthcare workforce and, subsequently, the quality of patient care. As country-level capacity to increase the number of healthcare workers in Mozambique, overall, is already stretched [32], correcting weaknesses in the TMG preparation will not be fast, nor should it be. Successful training and deployment of TMGs to provide quality care in rural and resource-constrained healthcare settings will be a time- and finance-intensive intervention.
